# Investigation of an ongoing cluster of tuberculosis (TB) associated with prisons, England

**DOI:** 10.1017/S0950268826101630

**Published:** 2026-05-18

**Authors:** Lucy Findlater, Mailis Maes, Nathan Post, Amith Philip, Lauren Ahyow, Chantal Edge, Martin Dedicoat, Tracey Langham, Suzi Coles, Esther Robinson

**Affiliations:** 1 UK Field Epidemiology Training Programme, UK Health Security Agency, UK; 2 Tuberculosis Unit, UK Health Security Agency, UK; 3 Health Protection in the Regions, UK Health Security Agency, UK; 4 Health Equity and Inclusion Health Division, UK Health Security Agency, UK

**Keywords:** Tuberculosis, Prisons, Disease Outbreaks, Whole Genome Sequencing, Communicable Disease Control

## Abstract

Prisons are high-risk environments for the spread of tuberculosis (TB). We investigated an ongoing prison-associated TB cluster to identify common exposures. We defined cases as residents in England with laboratory-confirmed TB belonging to the genetic cluster. We extracted records from the National TB Surveillance System, linked to prison operational data, and conducted descriptive analysis of demographic, clinical, and phylogenetic data. Years were masked for anonymity. Over six years, 12 individuals were identified, predominantly male (83%) and median age 34 years, in London, East Midlands, and East of England. Prison history was noted for 7/12 (58%) at 13 prisons. Everyone without prison history completed treatment, but 2/7 with prison history were lost to follow-up and 5/7 were still in treatment or outcome not evaluated. We identified frequent prison transfers and concurrent stays of four individuals at Prison B, and two at Prison G. Phylogenetic analysis indicated distinct sub-clusters with and without prison history, suggesting expansion of the cluster from community into prisons. Prisons continue to play a role in TB in England. Factors such as frequency of transfers could hinder treatment completion and control of transmission. Combining phylogenetic, epidemiological, and prison operational data facilitated investigation of transmission pathways.

## Background

Tuberculosis (TB) is a major global public health issue, with a quarter of the world’s population estimated to be infected with the causative agent *Mycobacterium tuberculosis*, and 1.5 million TB-associated deaths occurring per year [1, 2]. An estimated 225000 people fell ill with TB in the World Health Organization (WHO) European region in 2023, with a greater burden amongst the most vulnerable and impoverished population groups [2–4]. In England, TB rates are increasing, and 9.5 notifications of TB per 100000 population were recorded in 2024, close to the WHO threshold of a low-incidence country (<10 per 100000 per year) [5]. TB is a notifiable disease in England, with all individuals with suspected and confirmed TB required to be notified to the UK Health Security Agency’s (UKHSA) National TB Surveillance System (NTBS). Whole genome sequencing (WGS) of all isolates from individuals with culture-positive TB is conducted to identify genetically linked clusters of TB and potential transmission chains in the UK [6].

Prison residents experience higher rates of TB due to a combination of the overall poorer health of the prison population and characteristics of the prison setting itself [7]. These environmental factors include overcrowding, limited basic sanitation and housing infrastructure, poor ventilation, difficulty accessing health services (leading to delayed TB detection or inadequate treatment), difficulty contact tracing and a highly transient population, and difficulty implementing infection control measures, including access to adequate personal protective equipment (PPE) [8, 9]. Additionally, prisoners are more likely than the general population to possess risk factors for TB infection and disease, including substance misuse, homelessness, mental health problems, malnutrition, and co-infections such as human immunodeficiency virus (HIV) [10]. Measures are in place to detect and treat TB in prisons, such as screening of people arriving in prison with a verbal questionnaire on their symptoms [11]. However, these are not sufficient to identify all individuals with TB, which can lead to delayed diagnosis and subsequent outbreaks involving rapid, widespread transmission.

## Outbreak detection

UKHSA detected a genetically related cluster of TB in England which had been ongoing for over six years, affected multiple geographic regions, and was increasing in size. Several individuals in the cluster had been incarcerated in prisons across England prior to or during their diagnosis. We conducted an investigation of this cluster, aiming to describe demographic, clinical, and phylogenetic characteristics, detect any common exposures such as concurrent prison stays, and identify lessons learned from this cluster to be applied to future management of TB clusters in prisons.

## Methods

### Data sources

Since 2018, UKHSA sequences all *M. tuberculosis* isolates in England for rapid resistance profiling and to evaluate the relatedness of isolates. The Forest pipeline is used with a 12-single-nucleotide-polymorphism (SNP) difference cut-off to determine a genetic cluster [6]. Individuals in this cluster were defined as residents in England with laboratory-confirmed TB, with a bacterial isolate falling within the specified 12-SNP cluster. Individual records were subsequently linked to the NTBS and the national incident management system (HPZone), and demographic, clinical, and risk-factor information was extracted. Social risk factors including prison history were reported by individuals to clinical staff, who then inputted this information into NTBS. Further details were obtained from the local knowledge of colleagues within the national TB unit.

For individuals in the cluster who had reported a prison history, detailed information on their prison history was extracted from the Prison National Offender Management Information System (P-NOMIS), managed by His Majesty’s Prison and Probation Service (HMPPS). P-NOMIS is an operational database which contains prison movement data. Individuals were linked to their prison movement data using unique prison numbers (National Offender Management Service (NOMS) number). Prison numbers were obtained from the NOMS database using name, date of birth, and National Health Service (NHS) number, where not already known. Exact dates and locations of incarceration were extracted.

### Data analysis

We described individuals in the cluster by person, place, time, genetic relatedness of bacterial isolate, treatment outcomes, and social risk factors including prison history. The exact years of the cluster have been removed and replaced with chronological labels to protect the anonymity of individuals. The region of residence of each person was estimated using a residential postcode if available, or custodial institution postcode if not, for those with prison history. The residential postcode is obtained at the time of diagnosis. We produced a timeline to describe detailed prison histories and identify overlaps in incarcerations. Descriptive analysis was conducted in R version 4.2.1 [12].

## Results

### Description of cluster

**Figure 1.**
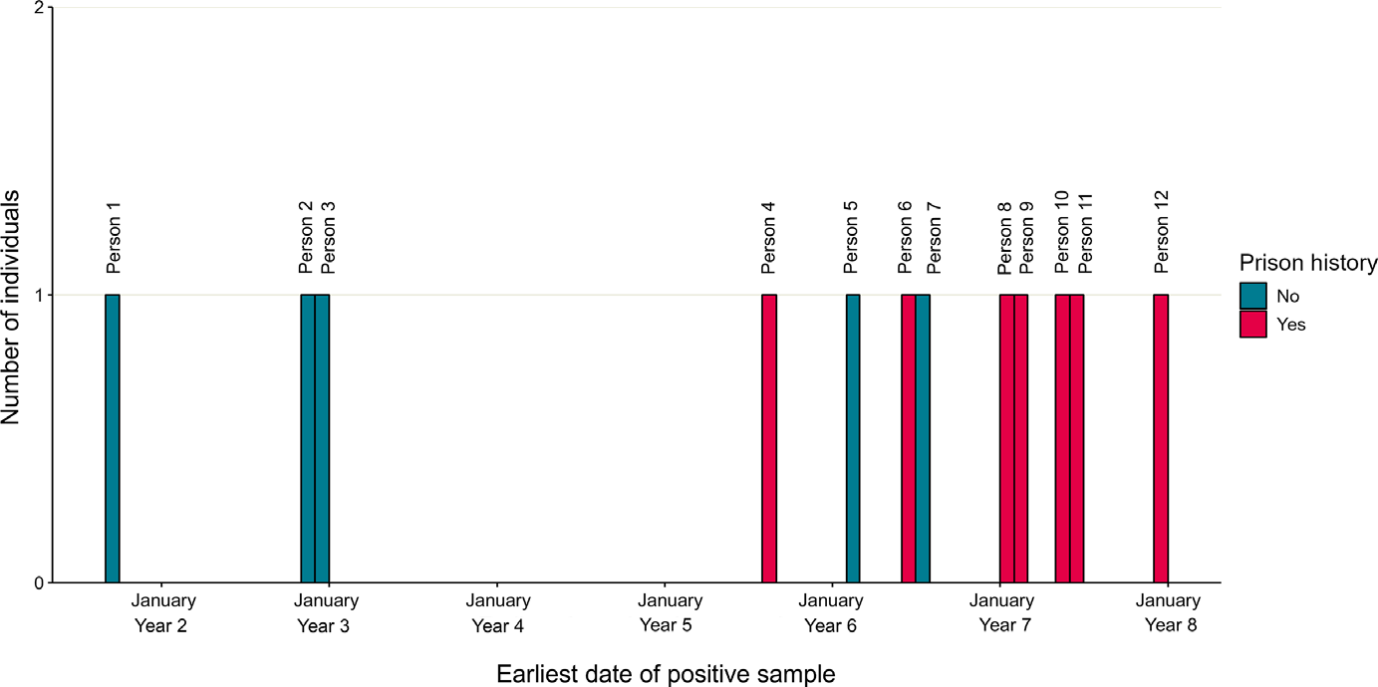
TB cluster described by date of earliest positive sample and prison history for each person. Graph showing the earliest positive sample date for each person in the cluster and whether the person had a prison history. The earliest positive sample from each person was as follows: sputum culture (4/12 people), sputum smear (4/12), pleural fluid culture (2/12), sputum polymerase chain reaction (PCR) (1/12), and bronchial tree histology (1/12). Years have been masked to protect anonymity and labelled with a year number instead. Sample dates range from September, Year 1, to December, Year 7. Figure 1. long description.

Over a six-year period, there have been 12 individuals identified in the cluster. The cluster was ongoing for over six years, with sample dates from September, Year 1 of the cluster, to December, Year 7 of the cluster (Figure 1). Individuals were mostly male (10, 83%), with a median age of 34 years, and resided in London [7], East Midlands [3], and East of England [2] (Table 1). The majority of individuals were born outside of the UK (9, 82%), and there was a range of countries of birth across Europe, Africa, and Asia.

**Table 1.** Characteristics of individuals within the TB cluster Table 1. long description.

Characteristic	Number of people (percentage)
Sex	
Male	10 (83%)
Female	2 (17%)
Age (years)	Median: 34 (Interquartile range: 27 to 38)
Age group (years)	
<20 years	1 (8%)
20–29 years	4 (33%)
30–39 years	4 (33%)
40–49 years	2 (17%)
50–59 years	0
60–69 years	1 (8%)
70+ years	0
Region	
London	7 (58%)
East of England	3 (25%)
East Midlands	2 (17%)
Country of birth	
Not UK-born	9 (75%)
UK-born	2 (17%)
Unknown	1 (8%)
Treatment outcome	
Completed treatment	5 (42%)
Still on treatment	5 (42%)
Lost to follow-up	2 (17%)
Prison history	7 (58%)

Table describing demographic characteristics and treatment outcomes of 12 individuals in the tuberculosis (TB) cluster, England.

### Prison history

**Figure 2.**
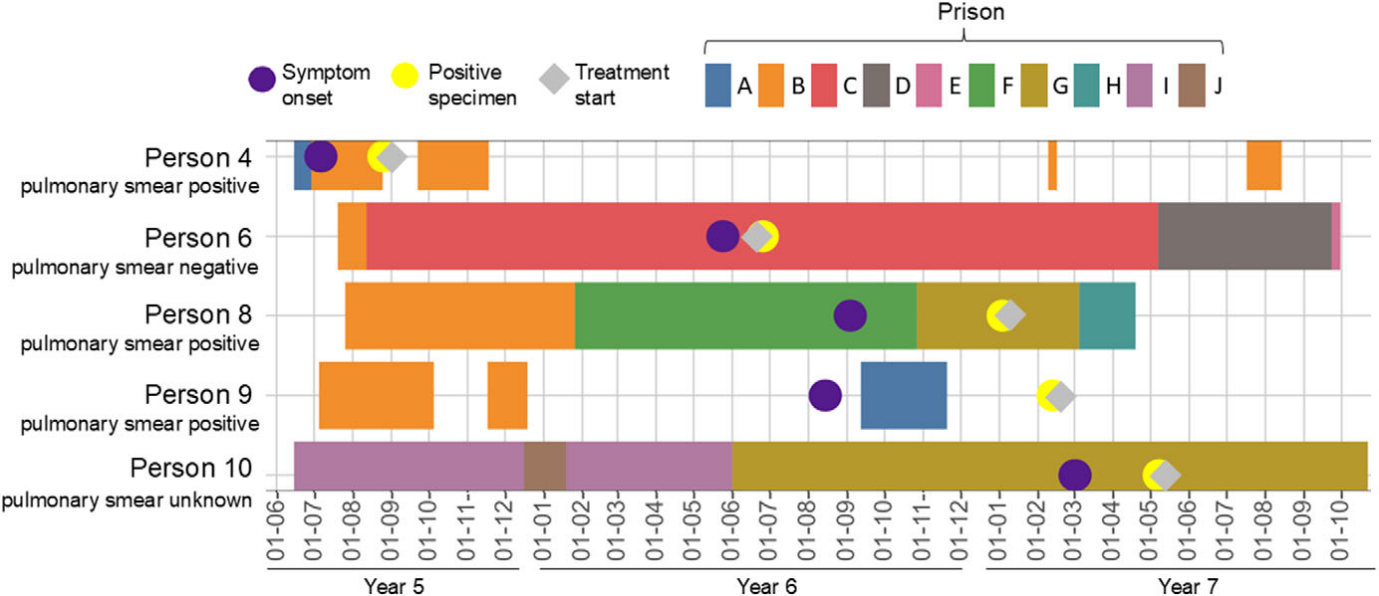
Timeline of prison stays, symptom onset date, and earliest positive sample date, of people in the TB cluster, where detailed prison history was available. Dates and locations of prison stays of people in the cluster, their symptom onset date, first positive sample date, and treatment start date are shown from June, Year 5, to October, Year 7. Years have been masked to protect anonymity and labelled with a year number instead. Of the 7/12 people in the cluster with prison history, 5/7 with detailed prison history information available are included in the timeline. Prison names are anonymized and labelled from A to J. Infectiousness is estimated using pulmonary smear result, either positive for *Mycobacterium tuberculosis*, indicating higher infectiousness, negative, or unknown. Figure 2. long description.

Overall, 7/12 (58%) individuals in the cluster had a prison history, with those with a prison history being identified more recently (Figure 1, Table 1). Overall, these seven individuals had been incarcerated at 13 prisons since the start of the cluster. Through linkage to the prison movements database, a detailed prison history was able to be obtained for 5/7 individuals with a prison history (Figure 2). The further 2/7 individuals who had a prison history were identified as belonging to the cluster after data linkage had been completed and could not be linked within the project timeframe. The data linkage revealed frequent movements of individuals between prisons, and an overlap of 4/5 individuals with data available at Prison B for 18 days, and 2/5 individuals at Prison G for just over four months (130 days). When combined with clinical information, we observed that the earliest-detected individual in the cluster, Person 4, had a positive pulmonary smear, indicating infectiousness, and a symptom onset date soon after arrival in Prison B. Persons 6, 8, and 9, who overlapped with Person 4 at Prison B, developed symptoms several months later. Person 8 had a positive pulmonary smear result and was symptomatic during their stay at Prison G, overlapping with the stay of Person 10, who later developed symptoms. All of these individuals started treatment up to three days after their first positive sample date, apart from Person 6, who started treatment two days before. However, they had all developed symptoms between 30 and 185 days (median: 69 days) before their treatment date, highlighting a transmission risk during their prison stays. Whilst we cannot determine the chain of transmission or identify missing links in the chain or possible transmission outside of prison, the overlapping prison stays are plausible epidemiological links between these individuals. Treatment outcomes for individuals with prison history were worse, with 2/7 lost to follow-up, 3/7 with a treatment outcome not evaluated 12 months after starting treatment, and 2/7 earlier in their treatment pathway, compared to 5/5 individuals without prison history completing treatment.

### Genetic relatedness

**Figure 3.**
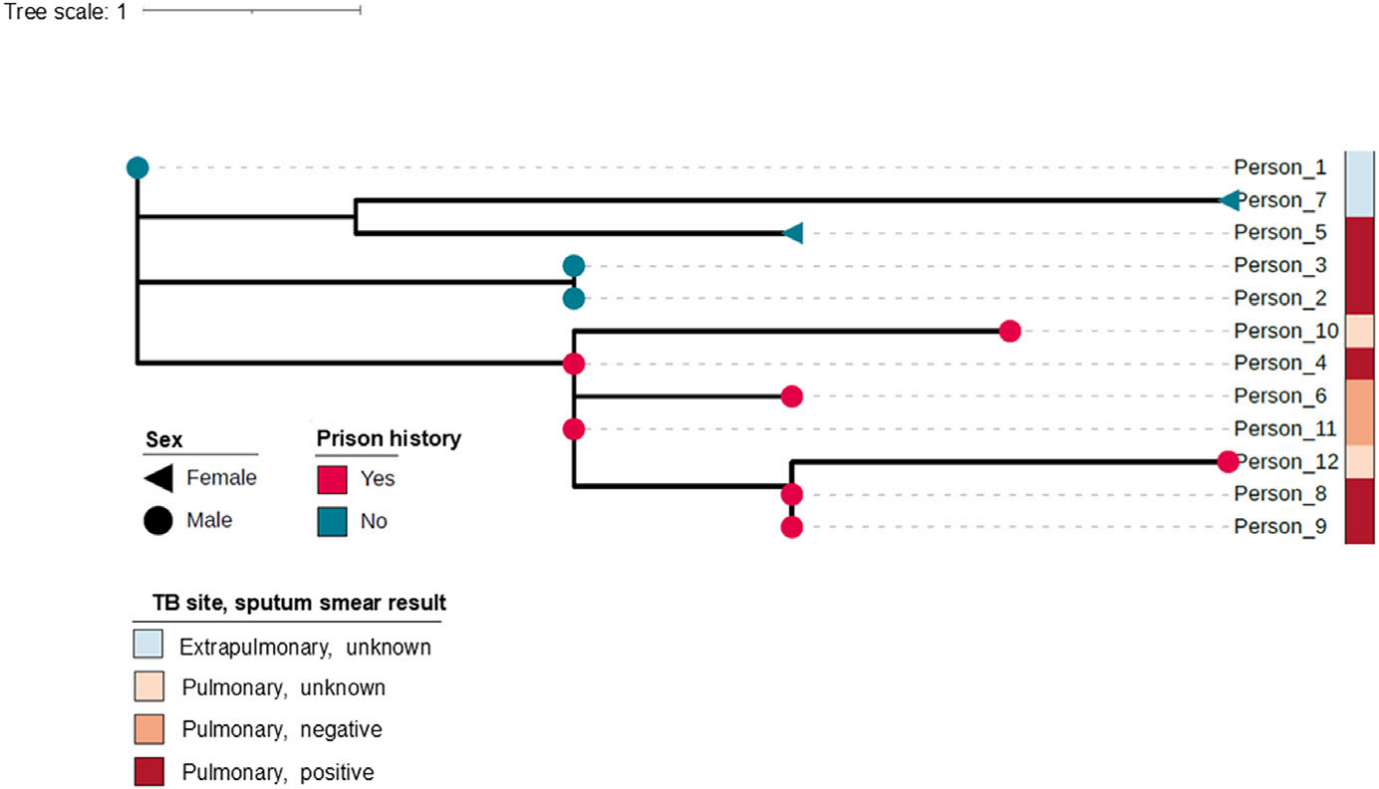
Phylogenetic tree showing genetic relatedness of *Mycobacterium tuberculosis* isolates from people in the cluster. Figure showing the genetic relatedness of *M. tuberculosis* isolates from the 12 individuals in the TB cluster. One isolate is shown per person as a dot in the tree, with the shape and colour of the dot showing the sex of the person and whether the person had a prison history. The tree scale represents 1-single-nucleotide-polymorphism (SNP) difference between isolates. Infectiousness of each person is estimated using pulmonary smear result, either positive for *M. tuberculosis*, indicating higher infectiousness, negative, unknown, or an extrapulmonary infection. Figure 3. long description.

Analysis of WGS data from isolates obtained from individuals in the cluster is shown in Figure 3. The phylogenetic tree suggests distinct sub-clusters of the earlier-detected individuals with no prison history and of the later-detected individuals with prison history. The earliest notified individual with a known prison link, Person 4, and Person 11 appear to be the most recent common ancestors for the prison-associated sub-cluster.

## Discussion

We described a genetically related, geographically dispersed TB cluster which was ongoing for over six years, with a more recent increase in the number of individuals detected and in the proportion who had been in prison prior to or during their diagnosis. Through linkage to prison operational data, we identified further overlaps of individuals with infectious TB at the same prisons which had been suspected but not confirmed during initial public health investigations. WGS data suggested distinct sub-clusters of the earlier-detected individuals without prison history and the later-detected individuals with prison history, who had closely genetically related bacterial isolates. Both the timeline of the cluster and the WGS data were suggestive of the introduction of this TB cluster into the prison network and subsequent transmission during overlapping prison stays. Our work highlighted the challenges of mapping and interrupting transmission in prisons, with the continuing detection of individuals in the cluster and limited evidence of treatment completion amongst those with prison history.

Prison populations are known to be at higher risk of TB in both high- and low-incidence countries, and this cluster highlighted the continued role of prisons in TB transmission in England [8, 13, 14]. Our investigation highlighted the frequency of prison movements, with individuals incarcerated at many sites over a short period of time. This brings challenges associated with identifying and tracing large numbers of contacts during prison stays (for example, frequently changing cellmates), transfers, and after release. We were not able to explore shared activities, cells, or wings of individuals overlapping in prisons, or control measures implemented for this cluster, as this would require further data collection from prison staff recalling events several years previously. However, another investigation identified over 3000 contacts amongst incarcerated residents and staff when investigating an outbreak of 25 individuals with TB in multiple prisons [15]. Given the barriers to effective contact tracing, the role of TB screening should not be neglected [11, 14, 16]. Current guidelines recommend verbal screening on prison entry, in which individuals are asked on arrival whether they have any symptoms of infectious TB [11]. However, this approach is limited due to patient factors (lack of disclosure), staff factors (insufficient training), resource factors (lack of capacity to screen), and disease factors (latent, pre-symptomatic infection). Screening with chest X-ray would provide more sensitive detection of TB in prisons, and indeed a chest X-ray for new prisoners who have not had a chest X-ray in the past six months is recommended within 48 h of arrival if possible; however, most prisons do not currently have these facilities available [17]. Given the high turnover of prisoners, optimizing screening on entry, whether verbal or X-ray, and subsequent isolation if positive, could be an effective approach to disrupting TB transmission [18, 19].

Importantly, frequent transfers and releases from prison can also hinder completion of TB treatment. Handovers of TB care for individuals are locally arranged between TB services and prisons [11]. Ensuring that clear protocols are in place between stakeholder organizations would be valuable to support continuity of care during and after release. It is worth noting that other risk factors more commonly experienced by prisoners affect treatment completion, such as physical or mental health conditions or substance misuse, and in our investigation, limited treatment completion in prisoners could also be associated with the incarcerated individuals having started their treatment process more recently or limited availability of information on their outcomes [20]. Prison authorities are typically notified of TB detections and clusters in their sites through information sharing between prison healthcare teams, HMPPS, and local and national public health teams [21]. Less resource-intensive ‘inform and advise’ activities during TB clusters could also be beneficial in encouraging symptomatic individuals to seek aid sooner, and clinicians to be alert to the possibility of TB in their patients [11].

Another challenge associated with frequent prison movements is difficulty recalling dates and locations of prison stays. We were able to overcome this issue by using prison records held by HMPPS, which provided accurate and detailed information on the movements of individuals between settings and filled in gaps in self-reported timelines. The prison database is the only system used by English and Welsh prisons, including private prisons, so it should include a record for everyone held in prison or transferred at that time. However, it is worth noting that, as with any large-scale administrative system, it is subject to possible data entry errors and reliant on information being held within the system at the date of extraction. Overall, combining custodial and epidemiological data greatly facilitated the investigation and highlighted the importance of knowledge sharing and cooperation between partner organizations. Revisiting this long-standing TB cluster using this approach allowed us to uncover new epidemiological links between individuals. However, given the long delay between the exposure events and our investigation, the transiency of prison populations, and that risk assessment and control measures such as contact screening had already been conducted at affected prisons, we did not recommend re-implementing further control measures for this cluster due to limited expected benefit and high resource requirement. Our investigation emphasizes the importance of rapid investigation of future TB clusters using WGS, custodial, and epidemiological data to establish transmission links, implement control measures in a timely manner, and justify the resource demand on prisons.

Additionally, the routine use of WGS for TB detections in England enabled detection and description of a geographically dispersed cluster. The investigation brought together distinct regional teams, which raised awareness of the cluster and encouraged information sharing. Other studies have documented the spread of TB between the general community and prison populations, which would be consistent with the timeline of this cluster [22]. These examples highlight the importance of preventative measures to stop the introduction and subsequent widespread transmission of TB within prisons.

## Conclusions and recommendations

Our investigation into a long-standing genetic cluster of TB highlights the continued importance of prisons in TB transmission in England. Through the use of prison operational data, we identified overlapping stays of individuals in the cluster at multiple prisons whilst at least one person was infectious, providing opportunities for transmission. The timeline of prison movements and WGS data are suggestive of introduction of this TB cluster into prisons from the community, followed by subsequent transmission within the prison network. We observed limited treatment completion and continued detection of individuals in the cluster within prisons. This could be partly associated with high frequency of prison transfers, which could complicate contact tracing and treatment access, and highlights the importance of optimizing screening on prison entry as an opportunity for TB detection and control. Given limited treatment completion, the implementation of protocols for prisons to support continuity of care during and after incarceration could be beneficial. Collaboration and data sharing between prison, medical, and public health partners were highly valuable to assess accurately common exposures in the cluster. We recommend rapid investigation of prison-associated clusters using custodial, epidemiological, and genetic data to identify links and implement control measures in a timely manner.

## Data Availability

Data were collected as part of a public health response and are considered sensitive and not publicly available.

## Long descriptions

### Figure 1 Long description

Graph showing the earliest positive sample date for each of the twelve individuals in the tuberculosis cluster, colour-coded by whether the person had a prison history (yes or no). Sample dates range from September, Year 1, to December, Year 7. Seven of the twelve people had prison history. Years have been masked to protect anonymity and have been labelled with a year number instead.

Navigate back to Figure 1.

### Table 1. Long description

The table describes demographic characteristics and treatment outcomes of the twelve people in the tuberculosis cluster in England. Overall, ten people (83%) are male and two people (17%) are female. The median age is 34 years with an interquartile range of 27 to 38 years. One person (8%) is aged under 20 years, four people (33%) are aged 20-29 years, four people (33%) are aged 30-39 years, two people are aged 40-49 years (17%), and one person (8%) is aged 60-69 years. Seven people (58%) reside in London, three (25%) in East of England, two (17%) in East Midlands. Five people (42%) completed their tuberculosis treatment, five people (42%) were still on treatment at the time of the study, and two people (17%) were lost to follow up. Seven people (58%) had prison history.

Navigate back to Table 1..

### Figure 2 Long description

Timeline showing the duration of each prison stay and the prison name for each of the five people with detailed prison history available. The timeline also shows the symptom onset date, first positive sample date, treatment start date, and sputum smear result for each person. Data are shown from June, Year 5, to October, Year 7. Years have been masked to protect anonymity and have been labelled with a year number instead. Prison names are anonymised and labelled from A to J. Each individual is labelled with their sputum smear result for Mycobacterium tuberculosis, categorised into pulmonary smear positive (three people), pulmonary smear negative (one person), or unknown (one person).

Navigate back to Figure 2.

### Figure 3 Long description

Phylogenetic tree showing the genetic relatedness of Mycobacterium tuberculosis isolates from the twelve individuals in the tuberculosis cluster. One isolate is shown per person as a dot in the tree, with the shape and colour of the dot showing the sex of the person (male or female) and if the person had a prison history (yes or no). The tree scale represents 1 single nucleotide polymorphism (SNP) difference between isolates. Each individual is labelled with their site of tuberculosis and sputum smear result for Mycobacterium tuberculosis, categorised into pulmonary TB with positive sputum smear, pulmonary TB with negative sputum smear, pulmonary TB with unknown sputum smear, and extrapulmonary TB.

Navigate back to Figure 3.
